# Effects of Commercial Antioxidants in Feed on Growth Performance and Oxidative Stress Status of Weaned Piglets

**DOI:** 10.3390/ani11020266

**Published:** 2021-01-21

**Authors:** Juan Orengo, Fuensanta Hernández, Silvia Martínez-Miró, Cristian Jesús Sánchez, Camila Peres Rubio, Josefa Madrid

**Affiliations:** 1 Department of Animal Production, Faculty of Veterinary Science, Regional Campus of International Excellence “Mare Nostrum”, University of Murcia, 30100 Murcia, Spain; nutri@um.es (F.H.); silviamm@um.es (S.M.-M.); cristianjesus.sanchez@um.es (C.J.S.); 2 Interlab-UMU, Faculty of Veterinary Science, Regional Campus of International Excellence “Mare Nostrum”, University of Murcia, 30100 Murcia, Spain; camila.peres@um.es

**Keywords:** oxidative stress, vitamin E, antioxidant, post-weaning period, piglets

## Abstract

**Simple Summary:**

Supplementation with high levels of vitamin E (Vit E) is usually recommended for diets used during the post-weaning (PW) period, when piglets show reduced growth rate and are more susceptible to disease. We tested two commercial antioxidants (AOX) in pre-starter and starter diets to evaluate the growth performance and oxidative status of weaned piglets. At the end of each feeding phase, growth data and serum samples were collected. Data analysed were body weight (BW), average daily gain (ADG), average daily feed intake, and feed conversion ratio (FCR). As oxidative stress indicators, total antioxidant capacity, total serum thiols, superoxide dismutase, glutathione peroxidase, thiobarbituric acid reactive substances (TBARS), and Vit E (α-tocopherol) were determined in serum. At the end of the study, cortisol and interleukin-6 were also determined, as well as TBARS and α-tocopherol concentrations in liver and muscle. The lowest BW, ADG, and FCR were found in piglets fed a low Vit E diet without AOX for the starter period. The α-tocopherol levels in serum and liver and differences among treatments were in agreement with the experimental design. Using AOXs or usual Vit E levels in feed was shown to be a key factor in maintaining optimal performance in the PW period.

**Abstract:**

This work aimed to evaluate the effect of adding two different commercial antioxidants (AOX) products to pre-starter and starter diets using low vitamin E (Vit E as DL-α-tocopheryl acetate) levels on the growth performance and oxidative stress of piglets for the first six weeks post-weaning (PW). They were sorted by initial body weight (BW: 6.175 ± 0.931 kg) and randomly allotted to four dietary treatments (with six replicates per treatment): a positive control (PC) and a negative control (NC) diet, with normal and low dose of vitamin E (80 and 15 mg kg^−1^, respectively), both without AOX; the other two experimental diets with a low dose of vitamin E (LVE) plus LOXIDAN VD100 (LVE + AOX1) or LOXIDAN E Ros (LVE + AOX2). Growth data were recorded, and blood samples were taken, at the beginning (day 0) and at the end of each feeding period: pre-starter and starter (at days 14 and 42, respectively). No differences among dietary treatments were found with respect to growth performance in the pre-starter period (*p* ≥ 0.05). However, at the end of the starter period, a lower BW was found in piglets fed the NC diet compared to the other dietary treatments. Differences in daily gain and feed conversion ratio were also found either for the starter period or when the whole period was considered (*p* < 0.05), whereby piglets fed PC or LVE diets supplemented with AOX showed better growth performance compared to piglets fed the NC diet. Regarding Vit E (α-tocopherol) serum levels, there were no differences among treatments at day 0; but the serum values of this vitamin decreased in LVE diets at 14 and 42 days, but not in the PC. On day 42, the highest levels of α-tocopherol in liver were also found in piglets fed PC (*p* < 0.05). Nevertheless, in general, from a metabolic point of view and after checking the serum biochemical profile of piglets, there were no differences in other oxidative stress markers (*p* ≥ 0.05). The results showed that the AOX products used were able to compensate for the lower Vit E supply with respect to growth performance in the starter phase. The use of AOXs or usual levels of Vit E in feed constitutes a key factor in achieving optimal growth performance of piglets in the PW period.

## 1. Introduction

Oxidative stress is harmful to animal health and may result in lower growth performance, disease, and even death [1]. It is the result of an imbalance between the generation of free radicals and the antioxidant capacity of animals [1,2]. Free radicals are constantly being generated from oxygen by many metabolic pathways. However, there is an efficient antioxidant system that eliminates generally excessive oxidative radicals and protects the organism against cell injury. This antioxidant system is formed by some endogenous antioxidant enzymes, such as superoxide dismutase, glutathione peroxidase, and catalase, and non-enzymatic components, such as vitamins E and C, carotenoids, and phenolics (considered as the main exogenous antioxidants) [2,3]. Moreover, oxidation processes are responsible for decreasing the nutritional value and palatability of feeds [4], with vitamin E being the major antioxidant in tissues, protecting cell membranes from attack by free radicals, and being considered the first defensive line against lipid peroxidation [5]. Thus, the intake of oxidized feed increases the number of free radicals in the animal.

Oxidative stress is influenced by dietary, social, and environmental factors and may decrease feed intake and performance, affecting redox status and increasing the risk of diarrhoea in pigs [6]. This is especially critical during the post-weaning period, when young animals show a decline in serum vitamin E concentration [7], a lower growth rate, and greater susceptibility to diseases [8]. At weaning, social and environmental stresses occur as piglets are separated from their mothers and moved to other facilities, whereas dietary stress is a result of changing from sow’s milk to solid feed (mainly plant-based) [9,10].

Adding antioxidants in small quantities to diets has been a common practice for a long time for the purpose of preventing or greatly retarding the oxidation of nutrients such as fats [11], alleviating the negative effects of peroxidised lipids [12]. Additionally, supplementation of different antioxidants is usually recommended for those diets used during critical periods in order to enhance the antioxidant system and reduce oxidative stress in livestock [13]. Some natural and synthetic antioxidants and/or commercial blends are available for use in the pig industry [14]. However, the effects of many commercial blends, the composition of which is not fully known as a result of marketing reasons and patent protections, have not always been sufficiently demonstrated in the scientific literature.

The aim of this work is to evaluate the effect on growth performance and oxidative stress of adding two different commercial antioxidant (AOX) products to pre-starter and starter diets using low Vit E levels in piglets for the first six weeks post-weaning (PW).

## 2. Materials and Methods

All the experimental procedures were carried out in accordance with the Ethics Committee of the University of Murcia (A13170502), following the European regulations (2010/63/EU Directive) for the protection of animals used for scientific purposes.

### 2.1. Animals and Facilities

A total of 120 non-castrated male piglets (Large-White) were used. The study was carried out at the Veterinary Farm of the University of Murcia (South-East Spain) for the first six weeks PW. Piglets were weaned at 28 days of age, and then, they were sorted by initial body weight (BW: 6.175 ± 0.931 kg) and randomly allotted to pens; five piglets were housed per pen (on a plastic slat floor), and they were reared under intensive conventional conditions and usual handling. Pens were assigned to one of the four dietary treatments (described below), with six pens per treatment. Each pen was provided with a standard feeder and a nipple drinker, where piglets were allowed ad libitum access to feed and water throughout the experiment.

### 2.2. Dietary Treatments and Feeding Program

Four dietary treatments were established with normal and reduced dosages of vitamin E (Vit E) in feed as follows: a positive control (PC) diet with a normal dose of Vit E (80 mg DL-α-tocopheryl acetate kg^−1^) and a negative control (NC) diet with a low dose of vitamin E (LVE) supplementation (15 mg DL-α-tocopheryl acetate kg^−1^), both without AOX; another two experimental diets with LVE plus commercial AOX1 (LOXIDAN VD100, dosage 150 g t^−1^) (LVE + AOX1), or AOX2 (LOXIDAN E Ros, dosage 300 g t^−1^) (LVE + AOX2). Kaesler Nutrition GmbH (Cuxhaven, Germany) provided the commercial antioxidant products used in this study. LOXIDAN VD100 is a formula based on three antioxidant substances: butylated hydroxytoluene (BHT), propyl gallate, and citric acid; LOXIDAN E Ros is a formulation of purely natural antioxidants: rosemary extract and a mixture of α, β, γ, and δ tocopherol extracts from natural origin (E306) and from vegetable oils (1b306(i)).

The feeding program was divided in two phases throughout the 6-week period: pre-starter (for the first 2 weeks) and starter (for the next 4 weeks). Within each feeding period, all diets were pelleted and formulated so as to be isoenergetic and iso-nitrogenous, based on digestible amino acids according to the recommendations of FEDNA [15]. All diets were manufactured by a commercial company (Alia, Lorca, Spain), and its basal composition is shown in Table 1. Pre-starter and starter feed samples were analysed for dry matter and crude protein using procedures of AOAC (Association of Official Analytical Chemists) [16] (Method 950.46 and Method 960.52, respectively).

### 2.3. Growth Performance

The following growth data were recorded at the beginning and at the end of each feeding period (pre-starter and starter): body weight (BW), average daily gain (ADG), average daily feed intake (ADFI), and feed conversion ratio (FCR). This latter was calculated by dividing ADFI by ADG for each period. Mortality was also noted daily.

### 2.4. Blood and Tissue Samples

Blood samples were taken from two piglets per pen at the beginning and at the end of each feeding period (days 0, 14, and 42). On day 0, piglets within each pen were chosen according to their BW as being close to the average weight of the pen; the same animals were subsequently bled over time. Whole blood samples (4 mL) were collected by jugular venipuncture into vacuum tubes without additives (Vacuette^®^, Greiner Bio-One International GmbH, Kremsmünster, Austria) for serum biochemistry. Within 20 min after collection, the samples were stored at 4 °C, and then centrifuged at 2000× *g* for 10 min. The serum was collected and immediately frozen at −20 °C until further analysis.

Total antioxidant capacity (TAC), total serum thiols (Thiol), superoxide dismutase (SOD), glutathione peroxidase (GPx), thiobarbituric acid reactive substances (TBARS), and Vit E (α-tocopherol) were determined in serum as biomarkers of oxidative stress (days 0, 14, and 42). Serum cortisol and interleukin-6 (IL-6) were also determined at the end of the study (on day 42).

Serum TAC was determined using a method based on the inhibition of the radical ABTS [2,2′-azino-bis(3-ethylbenzthiazoline-6-sulfonic acid)] by the sample, as previously described by Erel [18]. Thiol concentrations were measured according to the method described by Jocelyn [19] and modified by Costa et al. [20]. The activities of SOD and GPx in serum were determined using the commercial kits Ransod and Ransel (Randox Labs, Crumlin, UK), respectively. Levels of TBARS were obtained following the method described by Buege and Aust [21] using a microplate reader (Powerwave XS, Biotek instruments, Winooski, VT, USA). The α-tocopherol was analysed by HPLC using a previously described assay by Nirungsan and Thongnopnua [22]. Cortisol was analysed using a chemiluminescence system with commercially available kits (Immulite^®^, Siemens Healthineers, Erlangen, Germany). Finally, IL-6 was determined using Pig Interleukin 6 (IL-6) ELISA Kit of Cusabio Biotech (Wuhan, China) and analysed in an automated plate reader. All biomarkers of oxidative stress, except for α-tocopherol and TBARS, were analysed in an Olympus AU600 automated chemistry analyser (Olympus Diagnostica Europe GmbH, Ennis, Ireland). All methods showed an inter- and intra-assay imprecision lower than 15%.

At the end of the study (on day 42), two piglets per pen were slaughtered in order to take liver and muscle (musculus *longissimus dorsi*) samples. TBARS and α-tocopherol were also determined in both samples. For those analyses, approximately 0.5 g of each tissue sample (liver and muscle) was cut, weighed, and homogenised with an automatic homogeniser (Precellys Evolution, Bertin Technologies, Saint-Quentin, Yvelines, France) in 1:2 (weight/volume, *w*/*v*) phosphate-buffered saline [23]. Homogenisation was performed at 8800 rpm in two and three cycles of 20 s for liver and muscle, respectively, with a 30 s pause between cycles. After that, the samples were centrifuged at 10,000× *g* for 15 min at 4 °C. The supernatant was harvested in Eppendorf tubes and analysed.

### 2.5. Statistical Analysis

All data were analysed using the SPSS program (SPSS Inc., Chicago, IL, USA). Initially, a chi-square test was used to determine potential associations between dietary treatment and piglet mortality throughout the study period. Growth performance and serum data, which were only determined at 42 day (cortisol and IL-6), were analysed by one-way ANOVA. For analysis of the weight at the end of each feeding period, a covariance model (ANCOVA) was used, including the BW at the beginning of the period as a covariate. The antioxidant and stress parameters from the blood samples (where multiple data were available for each animal) were analysed using a mixed model, which took into account the effect of dietary treatment, sampling time (with three levels: at days 0, 14, and 42), and its interaction as fixed factors, as well as the effect animal as random effect. The pen and animal were considered to be the experimental unit for growth performance and serum data, respectively. All reported means are least square means, and pairwise comparisons of means were performed using the least significant difference (LSD) test. Pearson correlation coefficients were used to determine the relationship between ADG and ADFI within each feeding period. Additionally, linear correlations were calculated between concentrations of the serum biomarkers of oxidative stress for each sampling time. The significance level was set at *p* < 0.05.

## 3. Results

### 3.1. Diets and Growth Performance

Chemical analyses showed that all diets were manufactured correctly according to the estimated composition (Table 1). For crude protein, the analytical values were lower than the estimates, both in the pre-starter and the starter period, although the values were similar among the various dietary treatments (ranging from 174 to 176 g kg^−1^ and from 158 to 162 g kg^−1^, respectively).

The average BW of piglets at the beginning of the study was 6.175 kg, without differences due to dietary treatments in accordance with the experimental design (*p* ≥ 0.05) (Table 2). Piglets reached a final BW of about 10 kg at the end of the pre-starter period, with no differences among treatments (*p* ≥ 0.05). However, at end of the starter period, lower weights were found for the piglets fed the NC diet (21.404 kg) when compared with the other dietary treatments (*p* < 0.001).

The ADG for the pre-starter period was 0.274 kg d^−1^. No differences were found among dietary treatments for ADG (*p* ≥ 0.05). There were also no differences in ADFI in the pre-starter period (*p* ≥ 0.05), with feed intake being higher than 350 g d^−1^ for all treatments. In addition, there was a strong correlation between ADG and ADFI (r = 0.786; *p* < 0.01). For FCR, no differences were found among the dietary treatments (*p* ≥ 0.05).

For the starter period, or when the whole period was considered, there were differences for ADG among the treatments (*p* < 0.05). The lowest ADG was found in piglets fed the NC diet (0.416 and 0.365 kg d^−1^ for the starter and whole period, respectively). The lowest ADFI was found numerically in piglets fed the NC diet, but without reaching statistical significance (*p* ≥ 0.05). Moreover, strong positive correlations between ADG and ADFI were found both for the starter period (r = 0.894; *p* < 0.01) and for when the entire 6 weeks were considered (r = 0.909, *p* < 0.01). Finally, FCR was improved with LVE + AOX1 and LVE + AOX2 diets in comparison with the NC diet (*p* < 0.05) and maintained this ratio with respect to the PC diet both in the starter period and when the whole period was considered.

On the other hand, these results were not affected by health problems. In this regard, the mortality rate was 4.6% for the whole period, and no significant differences were found among treatments (χ^2^~*p*-value = 0.873).

### 3.2. Oxidative and Stress Status

Table 3 shows the effects of dietary treatment and sampling time on the serum biochemical profile indicators of the piglets throughout the experiment. With respect to the diet effect, the serum parameters evaluated were not significantly different among treatments (*p* ≥ 0.05), except for α-tocopherol (*p* < 0.001). For the latter, it was observed that piglets fed a PC diet reached higher values in comparison with other dietary treatments (0.381 vs. 0.232, 0.199, and 0.234 µg mL^−1^ for PC, NC, LVE + AOX1, and LVE + AOX2, respectively).

The sampling time significantly affected TAC, thiol, TBARS, and α-tocopherol values (*p* < 0.05), with the lowest levels being obtained at 14 d for TAC and thiol, and at 14 and 42 d for TBARS and α-tocopherol.

**Figure 1:**
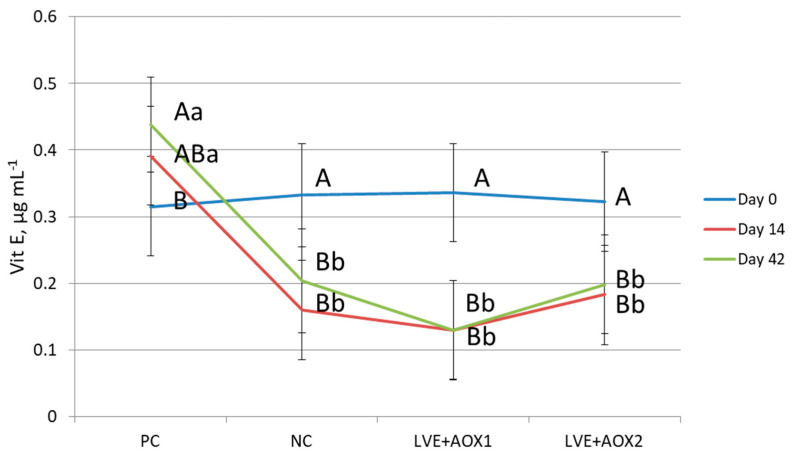
Effect of treatment by sampling day on vitamin E (α-tocopherol). Dietary treatments: positive control (PC) and negative control (NC) with normal and low-dose Vit E, respectively; low-dose Vit E (LVE) supplemented with LOXIDAN VD100 (AOX1) or LOXIDAN E Ros (AOX2). Means with different upper and lowercase letters within diets or sampling time, respectively, are significantly different at *p* < 0.05.

No interactions were found between diet and sampling time for most of the serum indicators tested, and there was only a significant interaction between both factors for α-tocopherol (*p* < 0.001). Indeed, α-tocopherol levels were not different among treatments at day 0; but the serum values of this vitamin decreased in the three LVE diets at 14 and 42 days, but not in the PC (Figure 1).

Pearson’s correlation coefficients (r) were calculated between concentrations of the serum biomarkers of oxidative stress for each sampling time (at days 0, 14, and 42) (Table 4). Regardless of the sampling day, consistent and strong correlations were found between TAC and Thiol, TAC and SOD, and Thiol and SOD. All these correlations were positive (*p* < 0.01) ranging from 0.765 to 0.902 between TAC and Thiol, from 0.532 to 0.792 between TAC and SOD, and from 0.569 to 0.938 between Thiol and SOD. No significant correlations were found between TAC and TBARS, Thiol and α-tocopherol, GPx and TBARS, GPx and α-tocopherol, or TBARS and α-tocopherol (*p* ≥ 0.05), whatever the day considered. The remaining combinations examined were not constant or repeatable over time; when their correlations were significant, the coefficient (r) was always lower than 0.5, except for SOD and α-tocopherol at day 0 (r = 0.547; *p* < 0.01).

At the end of the starter feeding period, two metabolites were added to the serum biochemical profile: cortisol and interleukin 6 (IL-6). The effects of the dietary treatments on these metabolites are presented in Table 5. For cortisol, no significant differences were observed among dietary treatments (*p* ≥ 0.05). The levels of IL-6 were not able to reach the minimum detection threshold (the threshold value was set at <1.25 pg mL^−1^). Moreover, the levels of TBARS and α-tocopherol observed in liver by treatment at 42 days were studied. TBARS values were significantly higher (*p* < 0.05) in the PC than other dietary treatments (6.42 vs. 5.05, 5.38, and 5.50 µmol g^−1^ for PC, NC, LVE + AOX1, and LVE + AOX2, respectively). Differences in α-tocopherol were also found (*p* < 0.05), with higher values being observed in piglets fed the PC diet (1.25 µg g^−1^) in comparison with the LVE + AOX2 diet (0.95 µg g^−1^), whereas the other treatments showed intermediate values for both groups.

Finally, the values of TBARS and α-tocopherol were also examined in the *longissimus dorsi* muscle. In this case, no significant differences were found in TBARS levels in the muscle among the treatments (*p* ≥ 0.05), while α-tocopherol did not reach detectable levels in this tissue for any treatment.

## 4. Discussion

Post-weaning is a challenging period for the health of piglets, influencing the redox status of animals. It has been shown that weaning is associated with a reduction in antioxidant mechanisms [24]. Moreover, an increase in oxidative products after weaning may affect the growth performance of newly weaned piglets [25]. Therefore, the addition of different commercial antioxidants to post-weaning diets is often recommended to protect animals from oxidative stress and maintain growth rates in the early post-weaning period.

In the current study, no differences were found with respect to growth performance (BW, ADG, ADFI, and FCR) among dietary treatments in the pre-starter period. However, with respect to the starter period, the growth performance in piglets fed low vitamin E plus antioxidant diets (LVE + AOX1 and LVE + AOX2) exhibited similar results to that of piglets fed the PC treatment (with a normal dose of Vit E), both of which were better than those piglets fed the NC diet (low Vit E group without adding antioxidants). In this feeding period, the AOX addition using low Vit E levels compared to the NC treatment increased BW (+14%), ADG (+21%), and decreased FCR (−11%).

It should be noted that many of the studies examining the effect of Vit E supplementation did not show significant effects on the post-weaning growth performance of piglets, regardless of whether they applied different doses, whether different sources of the vitamin were used, whether it was used in combination with vitamin C, or even whether it was tested using different administration methods (e.g., in water or in feed) [7,26,27,28]. On the other hand, some authors observed quantitative improvements in the growth performance of post-weaning piglets when Vit E levels were increased at moderate doses compared to a control group without Vit E [29]. This fact could be related to the short duration of the first post-weaning phases, since these significant effects were more frequently found in feeding trials covering the growing period [30,31]. Other authors [12] have suggested that the lacking or minimal effects at early ages may be due to the initial diets (pre-starter vs. starter) being composed of highly digestible and palatable ingredients. Our results showed that the lowest BW, ADG, and FCR were found in piglets fed low Vit E diet without adding AOX (NC diet), either for the starter period or when the whole period was considered. Likewise, Silva et al. [12] reported negative effects during the later feeding stages (from 21 d PW) on BW, ADG, ADFI, and gain: feed ratio through the addition of peroxidised soybean oil, where the supplementation with Vit E and polyphenols did not lead to significant improvements in growth performance despite the enhanced antioxidant capacity of the piglets. However, Lu et al. [13], who also supplemented diets high in oxidants with antioxidants, found that dietary addition of a blend providing ethoxyquin and propyl gallate (or this AOX blend + Vit E) was effective in improving growth. These results were not obtained when Vit E was applied alone. Along the same lines, our results showed that both commercial AOXs tested were able to compensate growth performance with respect to the PC diet in the starter phase despite the lower Vit E supply (LVE diets). Therefore, although both AOX products differed in their formulation, they showed similar effects on all the productive parameters, possibly through different metabolic pathways and mechanisms. With regard to their origin (and components) the two AOXs tested were based on synthetic (BHT, propyl gallate and citric acid) and pure natural (tocopherol and rosemary extract) antioxidants; both were without ethoxyquin. Currently, the European Union bans the use of ethoxyquin in the manufacturing of feeds for all animal species and categories [32]. Hence, it is also necessary to find an alternative solution.

At the beginning of the study, no differences were found among dietary treatments (day 0). In contrast, piglets fed LVE diets (including NC diet) showed low serum levels of this vitamin at the end of both feeding periods (at day 14 and 42) compared to piglets fed the PC diet (with a normal dose of Vit E). On average, the supplementation of Vit E increased serum levels by 72%, which is in agreement with the increase reported (85%) by Silva-Guillen et al. [12] compared with control and polyphenol treatments, although the inclusion levels tested and the serum concentrations of Vit E in that study were higher than those in our study. Therefore, the serum α-tocopherol levels and the differences found among the dietary treatments were in agreement with the experimental design. In this sense, Leskovec et al. [33], by adding olive leaf extract at different concentrations to oxidative stress-inducing diets compared to Vit E, found that piglets fed diets without Vit E supplementation had lower levels of plasma α-tocopherol in comparison with the supplemented group without affecting ADG. On the other hand, it is also known that the α-tocopherol concentration by itself shows a decline after weaning [7].

No effect was found on the other serum indicators sampled throughout the experiment as a result of diet (TAC, Thiol, SOD, GPx, TBARS, cortisol, and IL-6). In general, from a metabolic point of view, and after checking the serum biochemical profile of the piglets, our results did not show differences in terms of other oxidative stress markers that could explain the differences in growth performance among treatments. Regardless, the piglets in our study were expected to have lower basal oxidative stress-induced damage compared with other studies using pro-oxidant diets [12,13], other than that resulting from oxidative stress caused by weaning itself. Moreover, several authors found that the use of AOXs improved some of these indicators, although it was not always the same marker. This fact could be related to the type of diets and AOXs used, the different doses and mechanisms of action of each antioxidant applied, whether they were applied alone or in combination [12,33], or even the influence of some environmental and management conditions that can affect the growth of piglets and their health, as well as their impact on oxidative stress [25,33]. In fact, it is clear that interactions occur among all the AOXs, and they work in a complex network to recycle and regenerate one another [34]. On the other hand, the stability of the added Vit E seems to be affected by the destruction through oxidation processes. The addition of some AOXs may reduce these oxidation processes, resulting in increased availability of Vit E [35]. Along the same lines, despite the commercial antioxidants tested not leading to an increase in serum α-tocopherol, they were able to maintain the growth of weaned piglets, probably due to metabolic effects different from those of Vit E. Nevertheless, it is necessary to elucidate the mechanisms of action of both natural and synthetic antioxidants, while trying to understand the mechanisms behind oxidative stress [36,37].

In addition, the results of our study showed that only three out of 15 Pearson’s correlation coefficients for the serum biomarkers of oxidative stress (TAC, Thiol, SOD, GPx, TBARS, and α-tocopherol) were positive and strongly associated (higher than 0.5) regardless of the sampling time, whereas lower, non-repeatable correlations or no correlations were found for the remaining combinations. This low association between them would suggest that the mechanisms of oxidative balance and homeostasis are intricate and complex. Therefore, taking in mind that oxidative stress is not a singular metabolic event, other biomarkers could be considered to explain the effect of different types of antioxidants, or to accurately predict animal growth performance response [38].

Finally, the levels of α-tocopherol and TBARS were examined in the liver and the *longissimus dorsi* muscle. Both the hepatic α-tocopherol and TBARS levels were significantly higher in the PC group than in groups that received the other dietary treatments. In general, it was found that liver α-tocopherol concentration increased as the dietary Vit E increased [28,39]. Additionally, as the blood level of α-tocopherol increased, the liver appeared to retain more α-tocopherol than adipose or tissue muscle [7]. On the other hand, the higher levels of TBARS in the liver of piglets fed with a normal dose of Vit E (PC diet) were unexpected, as most authors reported either no effect or a decrease in malondialdehyde equivalents (usually reported as TBARS assay values) when antioxidants were added to the diet [40,41]. However, Lu et al. [13] found that the supplementation of Vit E alone could not prevent the negative effects of the oxidative stress-inducing diet, and piglets fed with this diet tended to have greater liver TBARS concentrations at the end of the study when compared with other diets containing an antioxidant blend (ethoxiquin and propyl gallate). The results for TBARS and α-tocopherol in the liver samples, although apparently contradictory, showed that the liver was the organ with the highest metabolic activity [42], and therefore, spot observations could be insufficient to obtain complete information regarding the multiple and complex metabolic pathways that take place in this organ.

The α-tocopherol did not reach a detectable level in muscle for any treatment, in contrast to results found in piglets [27] or growing-finishing pigs [43,44]. In addition, despite α-tocopherol not being detected, no differences were found among treatments for the TBARS levels in *longissimus dorsi* muscle. It should be taken into account that our study was carried out in young animals selected for lean growth, whereas Vit E is fat-soluble and accumulates mainly in fatty tissues. Therefore, the response of different tissues varied depending upon their metabolic activities. Along the same lines, Jensen et al. [39] reported lower levels of α-tocopherol as a response to dietary intake in muscle and brain when compared with other tissues, such as kidney fat, subcutaneous fat, and liver. Moreover, it was shown that α-tocopherol accumulation is muscle-dependent, with greater amounts in red muscles (M. *psoas major*) than in white muscles (M. *longissimus dorsi*) [45], as a result of muscle fibre types and the number of subcellular components (mitochondria and microsomes) [38].

It is remarkable that α-tocopherol levels in tissues also depend on supplementation time. In this sense, it has been reported that α-tocopherol levels generally increased over a 49-day supplementation period [46], while our study only covered the first 42 days PW. Thus, Jensen et al. [46] showed that porcine liver responded rapidly to dietary α-tocopheryl acetate intake, while muscle and adipose tissue responded at a slower rate, demonstrating that α-tocopherol concentrations in serum and liver reflect the immediate nutritional status of the animal, while levels in adipose and skeletal tissue reflect its long-term nutritional history [47].

## 5. Conclusions

The results showed that AOXs were able to compensate growth performance in the starter phase despite the lower Vit E supply. However, the mode of action could not be explained only with reference to the markers sampled for oxidative stress. The antioxidant products used in this trial (AOX1 and AOX2) can give greater flexibility with respect to the typically used levels of Vit E in order to maintain optimal growth performance in piglets during the PW period. Further studies covering subsequent feeding stages will also be needed to assess potential long-term effects.

## Figures and Tables

**Table 1:** Ingredients and composition of basal diets (as-fed basis).

Item	Pre-Starter	Starter
Ingredients, g/kg		
Corn	300.00	300.00
Wheat	329.60	414.40
Barley	50.00	50.00
Whey, sweet	50.00	-
Soybean meal (450 g CP/kg)	123.00	121.00
Hamlet Protein-300 (HP-300)	79.00	58.00
Soybean oil	15.00	12.00
Glucose	10.00	-
Monocalcium phosphate 16.5/22.7	7.50	8.00
Calcium carbonate	6.70	7.20
Salt	4.00	4.90
DL-Methionine	2.20	1.90
Liquid Lysine (500 g/kg)	10.20	10.00
L-Threonine	2.80	2.60
Premix ^1^	10.00	10.00
Calculated composition ^2^		
ME, MJ/kg	13.80	13.60
CP, g/kg	182.2	172.2
Ileal digestible AA, g/kg		
Lys	12.5	11.5
Met + cys	7.2	7.3
Thr	8.2	8.3
Trp	2.7	2.6
Calcium	6.9	6.8
Total phosphorus	5.4	5.3
Digestible phosphorus	2.2	2.2
Analysed composition ^3^, g/kg		
DM	916.3	905.5
CP	175.5	160.3

^1^ Supplied per kg of pre-starter and starter diets with normal (DL-α-tocopheryl acetate (3a700), 80 mg) and low dosages (DL-α-tocopheryl acetate (3a700), 15 mg) of vitamin E: vitamin A (3a672a), 12,000 IU; vitamin D3 (3a671), 2000 IU; vitamin K_3_ (3a710), 2 mg; vitamin B1 (3a821), 1.5 mg; riboflavin, 4 mg; vitamin B6 (3a831), 2.5 mg; vitamin B12, 0.025 mg; niacin (3a315), 25 mg; folic acid (3a316), 0.5 mg; biotin (3a880), 0.1 mg; choline chloride (3a890), 220 mg; pantothenic acid (3a841), 13 mg; vitamin C (3a300), 100 mg; Iron (II) chelate of the amino acid glycine (3b108), 7.7 mg; Mn (3b503), 40 mg; Zn (3b603), 120 mg; Fe (3b101), 120 mg; Cu (3b405), 150 mg; I (3b202), 0.65 mg; Se (E8), 0.25 mg; L-Valine (3c371), 1700 mg; L-Tryptophan (3c440), 800 mg; Endo-1,4-β-xylanase (EC 3.2.1.8), 200 FXU; 6-phytase (EC 3.1.3.26), 500 FYT. ^2^ According to FEDNA [17]. ^3^ Based on analyses in duplicate per dietary treatment and corresponding to the average of each feeding period.

**Table 2:** Body weight (BW), average daily gain (ADG), average daily feed intake (ADFI), and feed conversion ratio (FCR) by treatment in the pre-starter and starter feeding period.

		Diets ^1^				
Item	PC	NC	LVE + AOX1	LVE + AOX2	SEM ^2^	*p*-Value
No. of pens	6	6	6	6		
BW, kg						
Start of the study (at 0 day)	6.182	6.192	6.160	6.167	0.193	1.000
End of pre-starter period (at 14 day) ^3^	9.757	9.776	10.022	10.178	0.080	0.223
End of starter period (at 42 day) ^4^	24.941^a^	21.404 ^b^	24.053 ^a^	24.897 ^a^	0.241	0.000
Pre-starter period, 0–14 day						
ADG, kg day^−1^	0.260	0.263	0.283	0.291	0.007	0.384
ADFI, kg day^−1^	0.382	0.370	0.367	0.410	0.014	0.718
FCR, kg feed kg^−1^ gain	1.483	1.412	1.326	1.439	0.033	0.425
Starter period, 14–42 d						
ADG, kg day^−1^	0.540 ^a^	0.416 ^b^	0.492 ^a^	0.518 ^a^	0.011	0.004
ADFI, kg day^−1^	0.857	0.763	0.810	0.846	0.018	0.300
FCR, kg feed kg^−1^ gain	1.586 ^a^	1.836 ^b^	1.649 ^a^	1.632 ^a^	0.014	0.000
Whole period, 0–42 day						
ADG, kg day^−1^	0.447 ^a^	0.365 ^b^	0.423 ^a^	0.443 ^a^	0.009	0.023
ADFI, kg day^−1^	0.699	0.632	0.663	0.700	0.016	0.407
FCR, kg feed kg^−1^ gain	1.567 ^a^	1.733 ^b^	1.579 ^a^	1.589 ^a^	0.013	0.001

^1^ Dietary treatments: positive control (PC) and negative control (NC) with normal and low-dose vitamin E (Vit E), respectively; low-dose Vit E (LVE) supplemented with LOXIDAN VD100 (AOX1) or LOXIDAN E Ros (AOX2). ^2^ SEM: Standard error of the mean. ^3^ BW adjusted for differences in initial weight, being the regression coefficient for BW at 0 d (kg): 1.395 ± 0.093 (*p* < 0.001). ^4^ BW adjusted for differences in initial weight, being the regression coefficient for BW at 14 d (kg): 2.648 ± 0.280 (*p* < 0.001). ^a,b^ Means within a row with different letters are significantly different at *p* < 0.05.

**Table 3:** Effect of dietary treatment and sampling time on total antioxidant capacity (TAC), total serum thiols (Thiol), superoxide dismutase (SOD), glutathione peroxidase (GPx), thiobarbituric acid reactive substances (TBARS), and α-tocopherol in serum.

	Diets ^1^ (A)				Sampling Time (B)			SEM ^2^	*p*-Value		
Item	PC	NC	LVE + AOX1	LVE + AO2	Day 0	Day 14	Day 42		A	B	A × B
Sample size ^3^	36	36	36	36	48	48	48				
TAC, mmol L^−1^	0.395	0.382	0.406	0.381	0.399 ^a^	0.370 ^b^	0.404 ^a^	0.006	0.448	0.030	0.646
Thiol, mmol L^−1^	0.182	0.185	0.196	0.176	0.196 ^a^	0.170 ^b^	0.188 ^a^	0.004	0.402	0.002	0.214
SOD, U mL^−1^	1.39	1.32	1.75	1.13	1.48	1.35	1.37	0.171	0.690	0.823	0.199
GPx, U L^−1^	2457	2550	2571	2236	2505	2378	2482	50.8	0.082	0.251	0.058
TBARS, µmol L^−1^	3.41	3.07	3.32	3.22	3.61 ^a^	3.06 ^b^	3.10 ^b^	0.103	0.684	0.035	0.341
α-tocopherol, µg mL^−1^	0.381 ^a^	0.232 ^b^	0.199 ^b^	0.234 ^b^	0.327 ^a^	0.216 ^b^	0.243 ^b^	0.012	<0.001	<0.001	<0.001

^1^ Dietary treatments: positive control (PC) and negative control (NC) with normal and low-dose Vit E, respectively; low-dose Vit E (LVE) supplemented with LOXIDAN VD100 (AOX1) or LOXIDAN E Ros (AOX2). ^2^ SEM: Standard error of the mean ^3^ Sample size: 12 animals per treatment at day 0, 14, and 42. ^a,b^ Means within diets or sampling time with different letters are significantly different at *p* < 0.05.

**Table 4:** Correlation matrix between total antioxidant capacity (TAC), total serum thiols (Thiol), superoxide dismutase (SOD), glutathione peroxidase (GPx), thiobarbituric acid reactive substances (TBARS), and α-tocopherol for each sampling time (at days 0, 14, and 42).

	Thiol			SOD			GPx			TBARS			α-Tocopherol		
Sampling Time ^1^	Day 0	Day 14	Day 42	Day 0	Day 14	Day 42	Day 0	Day 14	Day 42	Day 0	Day 14	Day 42	Day 0	Day 14	Day 42
TAC	0.765 **	0.768 **	0.902 **	0.583 **	0.532 **	0.792 **	−0.093	0.276	0.367 **	0.173	0.131	0.156	0.498 **	0.056	−0.118
Thiol				0.569 **	0.813 **	0.938 **	−0.007	0.349 *	0.410 **	0.319 *	0.150	0.231	0.271	−0.041	−0.280
SOD							−0.409 **	0.266	0.466 **	0.091	0.121	0.286 *	0.547 **	−0.225	−0.205
GPx										−0.039	0.176	0.216	−0.262	0.196	0.020
TBARS													0.106	−0.180	−0.028

Note: “*” and “**” indicate significant correlation at <0.05 and 0.01 levels, respectively. ^1^ Sample size: 48 animals for each sampling time (*n* = 48).

**Table 5:** Effect of dietary treatment on cortisol and interleukin 6 (IL-6) in serum and on thiobarbituric acid reactive substances (TBARS) and α-tocopherol in liver and *Longissimus dorsi* muscle at day 42.

		Diets ^1^				
Item	PC	NC	LVE + AOX1	LVE + AOX2	SEM ^2^	*p*-Value
Sample size ^3^	12	12	12	12		
Serum						
Cortisol, µmol g^−1^	4.48	4.62	3.99	5.32	0.186	0.081
IL-6, pg mL^−1^	<1.25	<1.25	<1.25	<1.25	-	-
Liver						
TBARS, µmol g^−1^	6.42 ^a^	5.05 ^b^	5.38 ^b^	5.50 ^b^	0.161	0.029
α-tocopherol, µg g^−1^	1.25 ^a^	1.06 ^ab^	1.04 ^ab^	0.95 ^b^	0.038	0.023
*Longissimus dorsi* muscle						
TBARS, µmol g^−1^	1.46	1.83	2.32	1.87	0.259	0.712
α-tocopherol, µg g^−1^	<0.1	<0.1	<0.1	<0.1	-	-

^1^ Dietary treatments: positive control (PC) and negative control (NC) with normal and low-dose Vit E, respectively; low-dose Vit E (LVE) supplemented with LOXIDAN VD100 (AOX1) or LOXIDAN E Ros (AOX2). ^2^ SEM: Standard error of the mean ^3^ Sample size: 12 animals per treatment. ^a,b^ Means within a row with different letters are significantly different at *p* < 0.05.

## Data Availability

Data sharing is not applicable to this article.
